# Cysteine 893 is a target of regulatory thiol modifications of GluA1 AMPA receptors

**DOI:** 10.1371/journal.pone.0171489

**Published:** 2017-02-02

**Authors:** Lotta von Ossowski, Li-Li Li, Tommi Möykkynen, Sarah K. Coleman, Michael J. Courtney, Kari Keinänen

**Affiliations:** 1 Department of Biosciences, Division of Biochemistry and Biotechnology, University of Helsinki, Helsinki, Finland; 2 Turku Centre for Biotechnology, Åbo Akademi University and University of Turku, Turku, Finland; 3 A.I. Virtanen Institute, University of Eastern Finland, Kuopio, Finland; Institut d'Investigacions Biomediques de Barcelona, SPAIN

## Abstract

Recent studies indicate that glutamatergic signaling involves, and is regulated by, thiol modifying and redox-active compounds. In this study, we examined the role of a reactive cysteine residue, Cys-893, in the cytosolic C-terminal tail of GluA1 AMPA receptor as a potential regulatory target. Elimination of the thiol function by substitution of serine for Cys-893 led to increased steady-state expression level and strongly reduced interaction with SAP97, a major cytosolic interaction partner of GluA1 C-terminus. Moreover, we found that of the three cysteine residues in GluA1 C-terminal tail, Cys-893 is the predominant target for S-nitrosylation induced by exogenous nitric oxide donors in cultured cells and lysates. Co-precipitation experiments provided evidence for native association of SAP97 with neuronal nitric oxide synthase (nNOS) and for the potential coupling of Ca^2+^-permeable GluA1 receptors with nNOS via SAP97. Our results show that Cys-893 can serve as a molecular target for regulatory thiol modifications of GluA1 receptors, including the effects of nitric oxide.

## Introduction

AMPA receptors are tetrameric ion channels which mediate fast excitatory glutamate signaling in the central nervous system. AMPA receptor subunits, GluA1-4, share a modular structure consisting of extracellular N-terminal (NTD) and ligand-binding domains (LBD), a transmembrane ion channel domain (TMD) and a cytosolic C-terminal tail (CTD) [1,2]. Unlike the extracellular and membrane-associated domains, which have highly conserved sequences and structures, CTDs of AMPA receptor subunits are more diverse and therefore likely to contribute to many subunit-specific features of AMPA receptors.

The GluA1 subunit, together with GluA2, is a constituent of the majority of AMPA receptors in the nervous system [3–5]. However, it is also able to form homomeric Ca^2+^ permeable receptors [2,6,7]. GluA1 features an ~80 amino acid long cytosolic tail which provides binding sites for interacting proteins and carries a class I PDZ binding motif at the C-terminus. The cytosolic interaction partners identified to date include four PDZ domain proteins: SAP97 [8,9], mLin-10 [10], Shank3 [11] and sorting nexin 27 [12]. Of these, SAP97, a member of the PSD-95 family of membrane-associated guanylate kinase homologs (MAGUK), has been studied most intensively. Although the exact physiological role and regulation of the interaction are still unclear, the association of SAP97 with GluA1-containing AMPA receptors has been reported to regulate intracellular trafficking of GluA1 receptors [13], modulation by protein kinases [14,15], dendritic growth [16], and delivery of new AMPA receptors to synapses [17–20]. In addition to these PDZ binding motif-mediated interactions, the GluA1 CTD is engaged in non-PDZ interactions with cytosolic proteins, including 4.1N [21], reversion-induced LIM protein (RIL) [22], and cGMP-dependent protein kinase II [23]. These additional interactions have been linked to regulation of cellular trafficking of GluA1 receptors.

In recent years, it has become evident that glutamatergic neurotransmission is strongly influenced by redox conditions and that glutamate receptors can both give rise to and serve as molecular targets of reactive oxygen and nitrogen species. The redox status of extracellular cysteine residues in n-methyl-d-aspartate (NMDA) receptors regulates receptor activity, further, Ca^2+^ influx through NMDA receptor channels has been shown to trigger formation of nitric oxide. This is mediated by physical coupling of NMDA receptors with neuronal nitric oxide synthase (nNOS) via PSD-95, a synaptic scaffold protein and a close relative to SAP97 [24]. Nitric oxide thus formed acts via two main mechanisms: it stimulates guanylyl cyclase thereby increasing the formation of cyclic guanosine monophosphate (cGMP), and it triggers S-nitrosylation of reactive cysteine thiols in target proteins by a direct non-enzymatic mechanism [25,26]. Among the S-nitrosylated proteins are the NMDA receptor itself [27], PSD-95 [28], and AMPA receptor associated proteins N-ethylmaleimide sensitive fusion protein (NSF) [29] and stargazin [30]. These S-nitrosylation events have an inhibitory effect on NMDA receptor responses, consistent with a neuroprotective function [31,32]. On the other hand, increased S-nitrosylation promotes AMPA receptor activity via stimulating receptor delivery to cell surface [29,30].

In the present biochemical study, we have examined the functional role of a reactive cysteine residue, Cys-893 [33] in GluA1 CTD. Our results show that Cys-893 is involved in the regulation of GluA1—SAP97 interaction and acts as a target for S-nitrosylation. Moreover, we demonstrate that SAP97 is able to serve as a scaffold to bridge GluA1 AMPA receptor with neuronal nNOS. These results highlight the regulatory potential of thiol modifications and NO signaling in AMPA receptor function.

## Results and discussion

The cytosolic C-terminal tail of GluA1 contains several sites for posttranslational modifications and docking of interacting proteins, which modulate receptor function and cellular localization. Our earlier study with synthetic GluA1 peptides revealed a reactive cysteine residue, Cys-893, in GluA1 CTD which readily forms a disulfide bond with SAP97 under the conditions of *in vitro* binding experiments [33]. In the present study, we have examined the role of GluA1 Cys-893 on the SAP97 interaction with full-length molecules in live cells and examined its potential as a target for S-nitrosylation. Additionally, prompted by findings on stimulation of nitric oxide formation by Ca^2+^-permeable AMPA receptors [34–36], we have investigated the potential of SAP97 in serving as a physical link between nNOS and AMPA receptors, in analogy with PSD-95 –mediated association of NMDA receptor with nNOS.

### Functional role of GluA1 Cys-893

**Fig 1:**
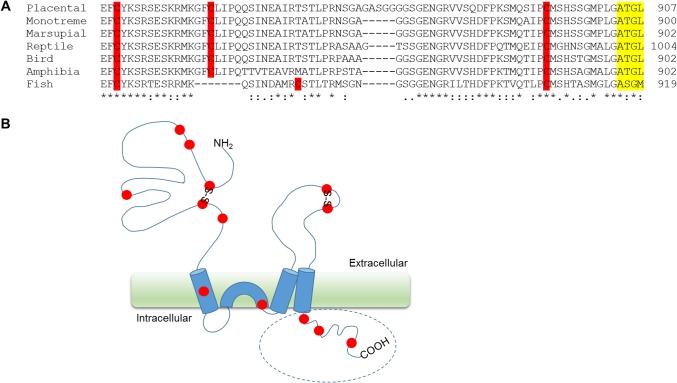
Conservation of cysteines in GluA1. (A) Sequence alignment of GluA1 C-terminal domains representing different vertebrate lineages: placental mammals (rat, *Rattus norvegicus*; accession number P19490), monotreme mammals (duckbill platypus, *Ornithorhynchus anatinus*; accession number F6YAX4), marsupial mammals (grey short-tailed opossum, *Monodelphis domestica*; accession number F7B4C1), reptiles (king cobra, *Ophiophagus hannah*; accession number V8PGM3), birds (chicken, *Gallus gallus*; accession number Q90855), amphibians (african clawed frog, *Xenopus laevis*; accession number B9V8R7), and fish (Mozambique tilapia, *Oreochromis mossambicus*; accession number Q90ZQ3). The sequences were aligned by using Clustal O (http://www.ebi.ac.uk/Tools/msa/clustalo/). Symbols below the alignment indicate the degree of sequence similarity: conserved residues (*), residues with strongly similar properties (:) and residues with weakly similar properties (according to Gonnet Pam250 matrix). Cysteine residues are highlighted in red and the PDZ binding motif in yellow. (B) Schematic drawing of the GluA1 subunit. Cysteine residues marked as red filled circles. C-terminal domain indicated with a circle.

Mammalian GluA1 subunits contain 13 cysteine residues, of which 8 are located in the extracellular domains, two in the membrane-associated segments and three in the cytosolic C-terminal tail (Fig 1). The three cytosolic cysteines, including Cys-893, are conserved in all land vertebrates, suggestive of functional importance. To examine the specific role of Cys-893 in GluA1, we replaced it with a serine residue. This minimal conservative change is not likely to introduce significant structural alterations to the wild-type (wt) protein, but should help reveal the thiol group–dependent functions.

For most of our experiments, we used N-terminally GFP-tagged constructs facilitating visualization of GluA1 transfected cells. Previously, N-terminally GFP-tagged GluA1 receptors have been shown to be functional and to exhibit similar trafficking to non-tagged receptor [37,38]. The GFP tag does not significantly influence the glutamate responses or the desensitization kinetics of homomeric GluA1 receptors in whole-cell patch clamp experiments (Figure A in S1 File). In addition, the extracellular N-terminal tag should not interfere with the molecular interactions of the cytosolic tails of the wild-type (wt) and mutant receptors.

**Fig 2:**
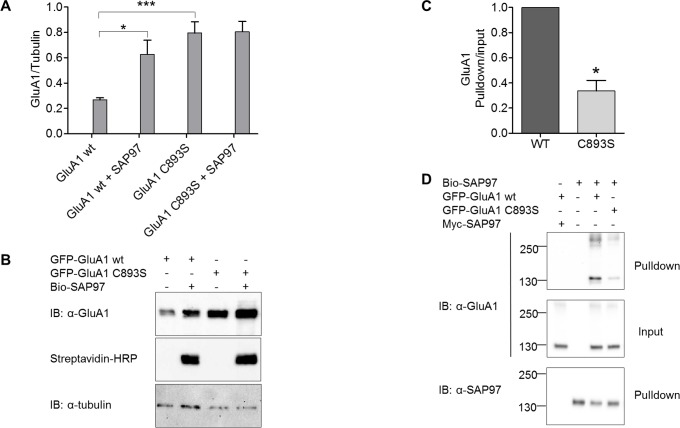
Role of GluA1 Cys-893 in SAP97 interaction. (A, B) Steady-state expression levels of GFP-tagged wt GluA1 and GluA1 C893S in HEK293T cells. (A) Immunoreactive GluA1 protein levels relative to tubulin (control for cellular protein) are shown for total cell extracts prepared from cells transfected for the expression of GluA1 or GluA1/Bio-SAP97. Equal amounts (10 μg) of GFP-GluA1 wt, GFP-GluA1 C893S, and Bio-SAP97 plasmids were used in the transfections; for single transfections, 10 μg of empty pcDNA3.1 plasmid was added to make the total amount of plasmid DNA 20 μg in all transfections. The data are expressed as mean ± SEM (*n* = 6), and were analyzed by one-way ANOVA with Bonferroni´s multiple comparison test. One asterisk (*) indicates P<0.05, whereas three asterisks (***) indicate P<0.001. (B) Representative immunoblots of total cell extracts. (C, D) Streptavidin pulldown assay showing the association of GFP-tagged wt GluA1 and GluA1 C893S with biotinylated SAP97. Plasmids encoding GFP-GluA1 wt, Bio-SAP97 or Myc-SAP97 plasmid were all used at 10 μg per transfection, whereas for GFP-GluA1 C893S only 3 μg plasmid DNA was used. The plasmid ratios for cotransfections were thus 1:1 for GluA1 wt + SAP97 and 3:10 for GluA1 C893S + SAP97. As above, the total amount of DNA was adjusted to 20 μg by adding empty vector where needed. In (C), the ratio of GluA1 band intensity in the pulldown to the intensity in the input is shown as mean ± SEM (n = 5); * indicates a P value of 0.0166 (one-sample t-test). (D) Representative immunoblots of detergent extracts (input) and streptavidin pulldowns. Statistical tests were performed using GraphPad Prism with a critical level of p < 0.05 used to determine statistical significance.

Wt GluA1 and GluA1 C893S receptors were expressed in transiently transfected human embryonic kidney cells (HEK293(T) cells), either alone or together with recombinant SAP97, a major cytosolic interaction partner of GluA1 AMPA receptors. In order to determine the physical association, SAP97 was expressed as an endogenously biotin-tagged molecule, obtained by fusion with *Propionibacterium shermanii* transcarboxylase [39,40]. Protein levels were estimated by quantitation of GluA1 western blotting signals normalized to tubulin, used as an internal control. Interestingly, when expressed alone (i.e., in the absence of SAP97), protein levels of GluA1 C893S were consistently 2–3 times higher than those of the wt receptor (Fig 2A and 2B). Co-expression with SAP97 led to an increased protein expression of wt GluA1, but had no influence on the C893S mutant (Fig 2A and 2B). These differences were similarly present both in the total cell extracts and in the Triton X-100 –soluble fraction; for the sake of clarity, only the total cell extracts are shown in Fig 2. These results suggest that the loss of thiol function leads either to increased protein stability (i.e. slower degradation rate) or to increased rate of synthesis of GluA1 receptors. We consider the latter possibility mechanistically unlikely because of the minimal difference between the wt and mutant constructs both at the amino acid and nucleotide sequence levels. These findings demonstrate that SAP97 is able to regulate GluA1 stability in a Cys-893 –dependent manner.

In contrast to increased protein levels of GluA1 C893S in the absence of recombinant SAP97, no difference was observed in the peak glutamate-triggered current amplitudes between the mutant and wt receptor channels in whole-cell patch clamp recordings, suggesting that despite its lower expression level, wild-type GluA1 channels reach the mature plasma membrane more efficiently than the C893S mutant (Figure B in S1 File).

To verify the effects of the C893S mutation on SAP97 interaction, indirectly suggested by the findings on protein levels, the association of the wt and mutant receptors co-expressed with biotin-tagged SAP97 was studied in a streptavidin pulldown assay. Due to the higher expression level of GluA1 C893S, we titrated the amounts of plasmid DNAs to produce similar total protein expression levels for the wt and mutant receptor. SAP97 complexes were harvested from cell lysate by the streptavidin pulldown assay and analyzed by western blotting. The blots verify that wt GluA1 and GluA1 C893S mutant were expressed at similar levels, and show that while both receptor forms were able to associate with SAP97, the mutation reduces the association with SAP97 (Fig 2C and 2D). Quantitation of the results from five separate experiments indicates a statistically significant and roughly 60–70% decrease in the association (Fig 2C). Streptavidin pulldown from cells expressing myc-tagged instead of biotin-tagged SAP97 did not produce any GluA1 immunoreactivity, consistent with specificity of the signals (Fig 2D). Although C893 is part of the C-terminal domain antigen in GluA1 detection, it is unlikely that possibly impaired immunoreactivity of C893S would significantly contribute to the observed reduced association of the mutant receptor with SAP97, because coassociation was measured as relative to the receptor present in the input sample, and because in western blot analysis the mutant and wild-type GluA1 do not show any noticable difference in immunoreactivity to anti-GluA1 antiserum (Figure D in S1 File).

The results described above indicate that while GluA1-SAP97 interaction can take place in the absence of Cys-893, the extent of the association can be modified by mechanisms which are dependent on Cys-893. The mechanisms which lead to reduced association of the mutant GluA1 with SAP97 are unclear at the moment, but a number of possibilities can be envisioned. Cys-893 in the free thiol form (or as a thiol conjugate) may promote an interaction-compatible conformation in GluA1 C-terminal domain, for example, via metal ion coordination or unique hydrogen bonding patterns. Alternatively, Cys-893 may directly participate in the interaction via bound metal ions or a disulfide bond. Interestingly, a disulfide forms readily between GluA1 C-terminus and SAP97 PDZ domain residue Cys-378 in binding experiments using purified proteins [33]. Even though disulfides are believed to be unstable in the cytosol, particular microenvironments in protein complexes may favor disulfides, and even transient, rapidly forming and breaking disulfides can be expected to have a stabilizing influence on the complex. Apart from the effects on SAP97 interaction, our results indicate that in the absence of SAP97, Cys-893 exerts a negative influence on the stability of GluA1, possibly by facilitating protein interactions or thiol modifications which enhance receptor degradation.

### S-nitrosylation of GluA1 and GluA1 CTD

**Fig 3:**
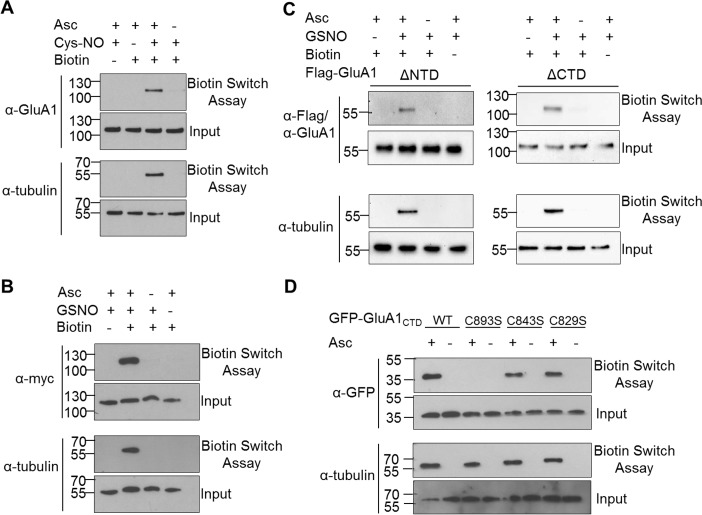
S-nitrosylation of GluA1. (A) HEK293 cells expressing myc-tagged full-length GluA1 were treated with 500 μM Cys-NO for 10 min, whereafter lysates were analyzed by the biotin switch assay to expose S-nitrosylated cysteines. (B, C) The susceptibility of GluA1 to NO was confirmed by treating HEK293 cell lysates expressing myc-tagged full-length GluA1 or flag-tagged deletion mutants thereof with GSNO and capturing S-nitrosylated GluA1 molecules through biotin switch assay. (D) GSNO- triggered S-nitrosylation of GFP-tagged C-terminal GluA1 wt and Cys point mutants analyzed by biotin switch assay. A positive signal on the biotin switch assay blots indicates the presence of S-nitrosylated cysteine residues. (A, B, C, D) Tubulin was used as a positive control for the biotin switch assay as it contains several cysteine residues sensitive to S-nitrosylation [42,61].

The strong effect of the C893S mutation on SAP97 interaction and on protein levels highlights the potential of Cys-893 in the functional regulation of GluA1. The underlying molecular mechanisms are still unclear, but considering the *in vitro* reactivity of C893, we propose that Cys-893 serves as a target of thiol-specific covalent modifications (Figure C in S1 File). S-nitrosylation is a physiologically important thiol modification and has been shown to play a role in the signaling and regulation of glutamatergic synapses [41]. Therefore, we analyzed whether GluA1, and specifically, its Cys-893 residue can be modulated by S-nitrosylation. For this purpose, we determined the receptiveness of wt GluA1 and its various deletion and point-mutated derivatives to S-nitrosylation by using the biotin switch assay [42]. Initially, S-nitrosylation was assessed in live HEK293 cells by briefly exposing transfected cultures to S-nitrosocysteine (Cys-NO), whereafter the cells were washed and lysed. After alkylation of free thiols, ascorbate was used to reduce S-nitrosothiols to free thiol groups, followed by conjugation to a thiol-reactive biotin derivative. As seen in Fig 3A, GluA1 contains one or more S-nitrosylated cysteines, which could be selectively reduced with ascorbate and biotinylated similar to tubulin, which was analyzed as a positive control (Fig 3A). The susceptibility of GluA1 to NO was confirmed by using nitrosoglutathione (GSNO) as the NO donor in HEK293 cell lysates, followed by a similar biotin switch assay (Fig 3B). In order to determine the location of the S-nitrosylated cysteines, we analyzed GluA1 receptors lacking either the large extracellular N-terminal domain (NTD) or the cytosolic CTD by biotin switch assay. S-nitrosylation of GluA1 was observed for both deletion mutants, suggesting that both extra- and intracellular parts of the receptor polypeptide contain cysteine residues susceptible to modification by NO (Fig 3C). Due to our interest in Cys-893, we examined the three C-terminal cysteines (Cys-829, Cys-843, and Cys-893) in more detail (Fig 3D). Biotin switch assay of wt and point-mutated CTDs (expressed as GFP fusion proteins) showed that C829S and C843S CTDs were S-nitrosylated similarly to wt CTD, whereas no signal was observed for C893S, indicating that Cys-893 is the only S-nitrosylation target in GluA1 CTD. These results are consistent with those by Selvakumar and coworkers, who reported, while this study was in progress, that Cys-893 of GluA1 is S-nitrosylated [43]. Somewhat differing from our results, their study suggested that all three CTD cysteines are S-nitrosylation targets, based on a slight decrease in the overall S-nitrosylation level observed for each individual single Cys-to-Ser point mutation introduced in GluA1 CTD. However, our results indicate that under similar experimental conditions, cysteines residing outside of the CTD are also S-nitrosylated, thus producing a significant background which makes it difficult to draw conclusions from the incremental decreases in S-nitrosylation. Our results extend the results of Selvakumar and coworkers by demonstrating that C893 is the dominant S-nitrosylated cysteine in GluA1 CTD and that cysteine residues located outside the CTD can also be S-nitrosylated.

### Association of GluA1 with nNOS

**Fig 4:**
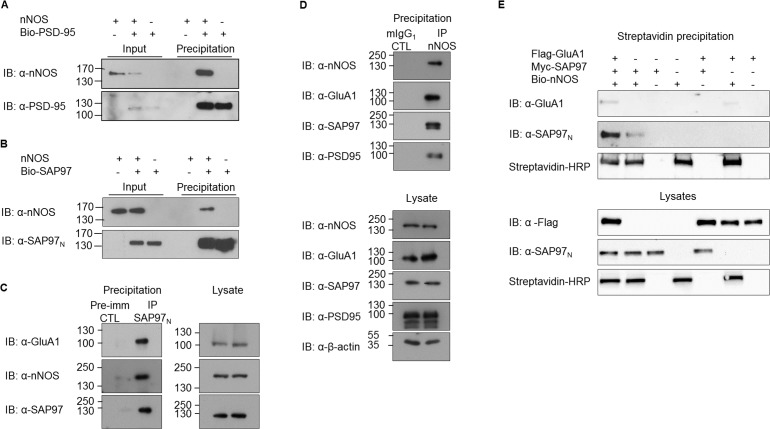
Association of SAP97 with nNOS. (A, B) Binding of nNOS to biotinylated MAGUK proteins PSD-95 (A) and SAP97 (B) shown in streptavidin pulldown assays. (C) GluA1 and nNOS co-immunoprecipitate with SAP97 in detergent extracts from postnatal day 7 rat cortex. SAP97 N-terminal domain–specific antiserum (SAP97_N_) was used for the immunoprecipitation, whereas the corresponding preimmune serum served as a control (CTL). The panel on the right shows the lysates. (D) GluA1, SAP97 and PSD-95 co-immunoprecipitate with nNOS in detergent extracts from postnatal day 7 rat cortex. Monoclonal nNOS antibody was used for specific immunoprecipitation, whereas monoclonal anti-MAP-2 served as control. Lysates are shown in the lower panel. (E) Streptavidin pulldown assay showing association of GluA1 to biotinylated nNOS. Immunoblots show representative images of experiments performed three or more times.

It is well-established that NMDA receptors are both coupled to and regulated by nitric oxide signaling, facilitated by the synaptic scaffold protein PSD-95, which bridges NMDA receptor-associated Ca^2+^ channels with neuronal nitric oxide synthase (nNOS) [24,44]. In addition, both colocalization and functional interactions between nNOS and AMPA receptors have been reported [34,35,45–49]. Having established GluA1 as an S-nitrosylation target and considering the functional parallels between the NMDA receptor and Ca^2+^–permeable GluA1 AMPA receptors, we examined whether SAP97, closely related to PSD-95, could link AMPA receptors with nNOS. First, we studied whether SAP97 is able to associate with nNOS, as suggested by an earlier report of coimmunoprecipitation of myc-tagged SAP97 and HA-tagged nNOS [50]. For this purpose, the SAP97 and PSD-95 (positive control) were expressed as biotin-tagged molecules and their association with nNOS was assessed by streptavidin pulldown. As expected, nNOS copurified with PSD-95 (Fig 4A). As seen from Fig 4B, nNOS copurified with SAP97 as well. No nNOS immunoreactivity was observed in the absence of biotinylated MAGUKs nor in the absence of nNOS expression, indicating the specificity of the signals (Fig 4A and 4B). Immunoprecipitation experiments performed with postnatal day 7 rat cortex confirmed the association of SAP97 with nNOS also in native tissue: nNOS was detected in SAP97 immunoprecipitates and SAP97 was present in nNOS immunoprecipitates (Fig 4C and 4D).

The ability of SAP97 to associate with both nNOS and GluA1 in native tissue (Fig 4C) suggests that, in principle, SAP97 may act as a scaffold bringing nNOS into the vicinity of GluA1. This notion is supported by the finding that nNOS immunoprecipitates contain not only PSD-95 and SAP97, but also GluA1 (Fig 4D). None of these molecules were detected in control immunoprecipitations performed with MAP2 antibody. To the best of our knowledge, this is the first report of a physical linkage between AMPA receptors and nNOS. Although coimmunopreciptation from brain extracts is consistent with the idea of a neuronal GluA1-SAP97-nNOS ternary complex, it does not prove it, and the result could also be produced by indirect linkages between GluA1 and nNOS. In order to test the formation of the ternary complex in a more defined system, biotin-tagged nNOS was expressed with Flag-tagged GluA1 and myc-tagged SAP97 in HEK293T cells. Streptavidin pulldowns from cellular detergent extracts (prepared two days after the transfection) were analyzed for the presence of GluA1 and SAP97. As seen in lane 1 of Fig 4E, which represents a sample from triply transfected cells, GluA1 is indeed co-precipitated with nNOS consistent with a ternary complex bridged by SAP97. Notably, in the absence of GluA1, SAP97 consistently showed less association with nNOS (lane 2, Fig 4E), suggesting that binding to GluA1 may promote further interaction of SAP97 with nNOS.

## Conclusions

**Fig 5:**
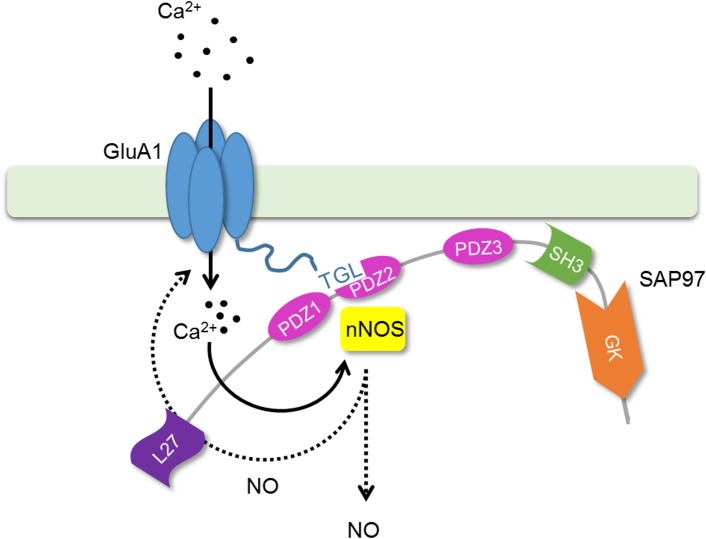
Cartoon model showing a ternary complex GluA1-SAP97-nNOS. GluA1 bound to SAP97 can associate with nNOS via PDZ-PDZ interaction [62]. SAP97 may exist as L27 domain–mediated dimers [18] or as heteromeric complexes with PSD-95 [54].

Our results demonstrate that a reactive cysteine residue, Cys-893, in the C-terminal domain of GluA1 is a target of S-nitrosylation, and potentially, other thiol modifications. Moreover, it has an important role in the regulation of protein stability and interaction with SAP97. Furthermore, we propose that GluA1 AMPA receptors can form SAP97-mediated complexes with nNOS to facilitate local control of (Ca^2+^-stimulated) NO production (Fig 5). The present findings underline the importance of thiol modifications in the dynamic regulation of glutamate signaling.

## Materials and methods

### DNA constructs

pcDNA3.1 (Thermo Fisher Scientific) -based plasmids for the expression of N-terminally Flag- or myc-tagged full-length GluA1 [9,51], its N-terminal domain deletion mutant consisting of residues 395–907 (ΔNTD) [52] and the pEGFP-C1 (Clontech) -based plasmid for the expression of N-terminally GFP-tagged GluA1 C-terminal domain consisting of residues 827–907, GluA1_ctd_ [53] have been described. Standard PCR-assisted cloning methods were used to prepare the following GluA1 expression plasmids: Flag-tagged GluA1 C-terminal domain deletion lacking residues 829–907 (ΔCTD) in pcDNA3.1, GFP-tagged GluA1_ctd_ mutants C829S, C843S and C893 in pEGFP-C1, and N-terminally GFP-tagged wt GluA1 and GluA1 C893S mutant in pcDNA3.1. In the latter two constructs, a segment encoding a signal peptide derived from GluA4 (residues 1–21; UniProtKB P19493) preceded the GFP sequence derived from pEGFP-C1. All GluA1 constructs represent the rat flip isoform (UniProtKB P19490).

The pcDNA3.1-based plasmids for the expression of N-terminally myc-tagged SAP97 and PSD-95 have been described [9,54]. Expression constructs for SAP97 and PSD-95 carrying N-terminal tags for *in vivo* biotinylation (Bio-SAP97 and Bio-PSD95, respectively) were constructed by replacing the segment encoding the N-terminal myc tag by a DNA fragment encoding the entire 1.3.S subunit of *Propionibacterium shermanii* transcarboxylase (i.e., biotinylation target domain; UniProtKB P02904; residues 1–123), obtained from the plasmid pEBB-PP [39] (a kind gift from Dr Kalle Saksela, University of Helsinki). SAP97 constructs represented the isoform i1b, i3. The expression construct for nNOS was constructed by inserting the human nNOS coding sequence (a kind gift from Dr Bernt Mayer, Karl-Franzens-Universität, Graz, Austria) to pcDNA3.1.

The correctness of all constructs was verified by restriction enzyme digestions and by sequencing through the PCR-derived parts.

### Antibodies and reagents

Primary antibodies used for immunoblotting and immunoprecipitation were as follows: rabbit anti-GluA1 antiserum recognizing the C-terminus of GluA1 (1:2000) [9], rabbit anti-SAP97_N_ antiserum recognizing the N-terminus of SAP97 (1:1000–2000) [9], anti-GFP mouse monoclonal antibody (1 μg/ml) (MBL International), anti-tubulin mouse monoclonal antibody (6 μg/ml) (Sigma), anti-FLAG M1 mouse monoclonal antibody (1 μg/ml) (Sigma), anti-NOS goat polyclonal antibody (0.5 μg/ml) (Sigma), anti-nNOS mouse monoclonal (0.4 μg/ml) (A-11, Santa Cruz Biotechnology), anti-myc rabbit polyclonal antibody (0.2 μg/ml) (Abcam), anti-SAP97 mouse monoclonal antibody (0.5 μg/ml) (K64/15, UC Davis/NIH NeuroMab Facility), anti-PSD95 mouse monoclonal antibody (0.5 μg/ml) (K28/43, UC Davis/NIH NeuroMab Facility), anti-GluA1 mouse monoclonal antibody (0.5 μg/ml, N355/1, UC Davis/NIH NeuroMab Facility), anti-β-actin mouse monoclonal antibody (0.1 μg/ml, 2D1D10, Genescript), anti-MAP-2 mouse monoclonal antibody (AP20, Leinco Technologies), anti-PSD-95 goat polyclonal antibody (0.4 μg/ml) (Santa Cruz Biotechnology). The secondary antibodies used were horseradish peroxidase conjugated anti-mouse, anti-rabbit (1:3000–8000) (GE Healthcare), anti-mouse (1:25,000) (Santa Cruz Biotechnology) or anti-goat IgG (1:3000) (Abcam). Horseradish peroxidase conjugated streptavidin (1:20,000) (Molecular Probes) was used for detection of biotin-labelled proteins in Western blots. PageRuler Plus Prestained Protein Ladder (Thermo Scientific) was used as size marker. Reagents used in the biotin switch assay, neocuproine, methylmethanethiosulfonate (MMTS), sodium ascorbate, glutathione, S-nitrosoglutathione (GSNO) and sodium deoxycholate (SDC) were purchased from Sigma. S-nitrosocysteine (Cys-NO) was prepared as follows: equimolar amounts of L-cysteine (diluted in 0.5 M HCl) and sodium nitrite (NaNO_2_) were mixed thoroughly for 1–2 min at RT protected from light. The reaction mixture was neutralized with NaOH and the concentration of the synthethized S-nitrosocysteine was calculated by measuring the absorbance at 543 nm (ε = 16.8 M^-1^ cm^-1^) [55,56]. Pyridyldithiol-biotin (biotin-HPDP) was purchased from Thermo Scientific. Molecular biology enzymes were from New England BioLabs or Thermo Scientific.

### Cell culture and transfection

HEK293 and HEK293T cells were grown in Dulbecco’s modified Eagle’s medium (DMEM) (Sigma-Aldrich) supplemented with 3.7 g/l sodium bicarbonate, 10% (v/v) fetal calf serum, HEPES, penicillin (50 units/ml), and streptomycin (50 units/ml) at 37°C under 5% CO_2_. HEK293 and HEK293T cells were transiently transfected by using calcium phosphate co-precipitation. The cells were harvested and used in streptavidin precipitation or biotin switch assays 40–50 h later.

### Streptavidin pull-down assay

Transfected HEK293 or HEK293T cells were lysed in TNE buffer (1% Triton X-100, 50 mM Tris-HCl pH 7.5, 120 mM NaCl, 1.0 mM EDTA, 1.0 mM NaF, 1.0 mM Na_3_VO_4_, 1.0 mM PMSF, 10 μg/ml each of leupeptin and aprotinin) and centrifuged at 16,000 g for 15 min at 4°C in a microcentrifuge. The supernatant was collected and a small aliquot was set aside, representing the total Triton-X-100 soluble protein pool (input). The rest of the supernatant was incubated 2 h or overnight at 4°C with streptavidin-sepharose (GE Healthcare) in order to capture biotinylated proteins and their interacting protein partners. Streptavidin-sepharose beads were collected by centrifugation at 500 g for 2 min at 4°C, washed two to four times with TNE buffer and then rinsed with minimal TNE (50 mM Tris-HCl pH 7.5, 120 mM NaCl, 1.0 mM EDTA). Proteins bound to streptavidin-sepharose were eluted in SDS-PAGE sample loading buffer and analyzed by Western blotting.

### Preparation of total protein cell extracts

Detergent extracts representing the total cellular protein level were prepared by resuspending transfected HEK293T cells in a modified TNE buffer containing sodium dodecyl sulphate (SDS) and sodium deoxycholate (SDC) (0.5% SDS, 1% SDC, 1% Triton X-100, 50 mM Tris-HCl pH 7.5, 120 mM NaCl, 1.0 mM EDTA, 1.0 mM NaF, 1.0 mM Na_3_VO_4_, 1.0 mM PMSF, 10 μg/ml each of leupeptin and aprotinin), whereafter the cell suspension was sonicated briefly and the suspension was mixed for 1–2 h at 4°C followed by a centrifugation at 20,000 g for 15 min at 4°C in a microcentrifuge. The supernatant containing the total cellular protein pool was collected.

### Biotin switch assay to analyze protein S-nitrosylation

The biotin switch assay was performed essentially as described previously earlier [42,57]. Briefly, transfected HEK293 cells were lysed in HEN buffer (250 mM Hepes, pH 7.7, 1 mM EDTA, 0.1 mM neocuproine) containing 1% Triton X-100, 0.1% SDC, 0.1% SDS, 1mM Na_3_VO_4_, 1.0 mM PMSF, 10 μg/ml each of leupeptin and aprotinin. Susceptible cysteine residues were nitrosylated in vitro by incubating cell lysates in 100 μM GSNO for 1 h RT in the dark. Excess GSNO was removed from the sample with Econo-Pac 10 DG desalting columns (Bio-Rad) using minimal elution protocol. Free thiols were blocked by methyl MMTS. Unreacted MMTS was removed by acetone precipitation (−20°C) and precipitated protein samples were resuspended in HEN buffer containing 1% SDS. S-nitrosylated cysteine residues were converted to free thiols with sodium ascorbate (50 mM final concentration). The free thiols were then biotinylated with biotin-HPDP at 25°C for 1–2 h. Proteins were precipitated with acetone (−20°C), and the pellet was resuspended in a 1:10 dilution of HEN buffer containing 1% SDS. Three quarters of a volume of Neutralization buffer (20 mM Hepes, pH 7.7, 100 mM NaCl, 1 mM EDTA, 0.5% Triton X-100) was thereafter added to the resuspended protein samples. Biotinylated proteins were captured overnight at 4°C with streptavidin-sepharose (GE Healthcare). Streptavidin-sepharose was collected by centrifugation and bound proteins were washed 4 times with high salt (600 mM NaCl) Neutralization buffer and finally eluted from the beads with Neutralization solution containing 100 mM 2-mercaptoethanol. Eluted proteins were analyzed by Western blotting. To study endogenous S-nitrosylation in transiently transfected HEK293 cell cultures, cells growing in dishes were exposed to Cys-NO for 10 min at 37°C, the cells were then washed with PBS and lysed in HEN buffer. Protein sensitivity to NO was assessed by the biotin switch assay as described above, starting with the blocking of free thiols with MMTS in the cell lysates.

### Co-immunoprecipitation

Co-immunoprecipitation was performed essentially as described earlier [58]. Cerebral cortical tissue from postnatal day 7 (P7) rats was homogenized in low-salt buffer (based on Cao et al. [59], with minor modifications: 20 mM Na_2_ β-glycerophosphate, 30 mM NaF, 2 mM EDTA, 10 μg/ml aprotinin, leupeptin, and pepstatin A, 0.1 mg/ml PMSF, 0.5% igepal), and precleared at 20,000 g for 10 min at 4°C. Immunoprecipitating (IP) antibody, nNOS (A-11, 2.5 μg/ml) or SAP97 antiserum (5 μl/ml) was added to the lysate. An irrelevant mouse monoclonal antibody (MAP-2, AP20, 2.5 μg/ml) was used as a control for nNOS IP and pre-immune serum (5 μl/ml) for SAP97 IP. Samples were rotated for 2 h at 4°C, whereafter 5 μl of protein-A magnetic beads was added, and rotation continued for 1 h. Beads were then washed three times with the low-salt buffer, and protein was eluted from drained beads by boiling at 95° for 10 min in SDS-PAGE sample loading buffer and analyzed by Western blotting [60].

### SDS-PAGE and immunoblotting

Proteins were electrophoresed in ready-made SDS-PAGE gradient gels from Bio-Rad (4–15%) and Lonza (4–12%), and electroblotted to polyvinylidene difluoride (PVDF) membranes (GE Healthcare). The membranes were blocked with 3–5% dry milk powder/TBS-Tween (or with 5% BSA/TBS-Tween for detection with Horseradish peroxidase conjugated streptavidin), incubated with the primary and secondary antibodies described above, and developed with Clarity Western ECL Substrate (Bio-Rad) or SuperSignal West Pico Chemiluminescent Substrate (Thermo Scientific). The chemiluminescent signals were detected by exposure to HyperfilmTM (GE Healthcare) or by the Bio-Rad ChemiDoc XRS system. Intensities of the bands were quantified by using the Bio-Rad ChemiDoc XRS system and Quantity One software.

### Ethics statement

According to the Finnish Act on the Use of Animals for Experimental Purposes (62/2006) and corresponding EU guidelines, tissues used in this study did not involve any animal experiment, and the specific use of animal tissue for the experiments included in this study were approved by the Director of the The University of Eastern Finland Lab Animal Centre with usage plan number EKS-003-2013.

## Supporting information

S1 File Supporting Information. (PDF)
