# Supplementary material for: Cysteine 893 is a target of regulatory thiol modifications of GluA1 AMPA receptors
Source: PLoS One. 2017 Feb 2;12(2):e0171489. doi: 10.1371/journal.pone.0171489 (PMC5289633; doi:10.1371/journal.pone.0171489)
Supplement: S1 File — (PDF) [file pone.0171489.s001.pdf]

Supporting Information

Materials and Methods

Electrophysiology

For patch clamp recordings, HEK293 cells were transfected with expression plasmids (1 μg/ml each) for N-terminally GFP- or myc-tagged GluA1 wt or GFP-tagged GluA1 C893S point mutant. Some of the transfections were performed as co-transfections with an expression plasmid encoding N-terminally biotinylated SAP97. EGFP (Enhanced green fluorescence protein; 0.5 μg/ml) was included in the transfections for visual identification of transfected cells. Whole cell patch clamp recordings were made from GFP-positive HEK293 cells with an Axopatch 1B patch clamp amplifier and pClamp 10 software (Molecular Devices, Sunnyvale, CA) at a holding potential of −60 mV. After gigaseal formation and whole cell configuration was achieved, by rupturing the cell membrane, the cells were lifted from the bottom of the dish to facilitate solution exchange. Electrodes were pulled from borosilicate glass capillaries (World Precision Instruments, Stevenage, UK) and had a resistance of 2–4 MΩ when filled with internal solution containing 140 mM CsCl, 2 mM $MgCl_2$, 10 mM EGTA, and 10 mM HEPES (pH adjusted to 7.2 with CsOH and osmolarity adjusted to 315 mosmol). Cells were continuously perfused with the recording solution containing 150 mM NaCl, 2.5 mM KCl, 2.5 mM $CaCl_2$, 1 mM $MgCl_2$, 10 mM glucose, and 10 mM HEPES (pH 7.4; 320 mosmol). 10 mM L-glutamate (Sigma-Aldrich) was diluted to the recording solution and applied to the cells using a piezo-driven applicator (Siskiyou piezo switcher, Siskiyou Corp., Grants Pass, OR). The time-constants of desensitization (tau values) were obtained by least-squares curve fitting to a single exponential function, $F(t) = A \exp(-t/tau) + C$, in Clampex 10.2 analysis program (Molecular Devices, Sunnyvale, CA, USA). In the equation, F(t) is the current amplitude at time t, A is the intial amplitude, and C is a constant to account for residual current. The graphs were prepared by using Prism 4.02 software (GraphPad, San Diego, CA). Each transfection was

done at least twice and 5–16 cells were recorded in each experiment. The data in the graphs are presented as mean ± SEM.

Western Blot Analysis of Immunoreactivity

To compare the immunoreactivity of the rabbit anti-GluA1 antiserum to wild-type GluA1 and GluA1 C893S point mutant, lysates containing GFP-tagged wt and mutant GluA1 were resolved in duplicates by SDS-polyacrylamide gel electrophoresis and transferred to PVDF membrane. After blocking the nonspecific binding by bovine serum albumin, the membrane was cut to strips and incubated either with rabbit anti-GluA1 antiserum (1:2000) targeting the C-terminal tail of GluA1, or with rabbit polyclonal anti-GFP antibody (1:1500) (Fitzgerald Industries International, RDI-GRNFP4abr), targeting the N-terminal GFP-tag. In addition, the strips were incubated with mouse monoclonal anti-tubulin antibody (1:1000) (Sigma) which served as a control for cellular protein. After washes, the bound antibodies were deteted by incubating with horseradish peroxidase conjugated anti-rabbit (Ge Healthcare) or anti-mouse (1:25,000) (Santa Cruz Biotechnology) IgGs, followed by chemiluminiscence reaction with Clarity Western ECL Substrate (Bio-Rad) and imaging by Bio-Rad ChemiDoc XRS system and Quantity One software.

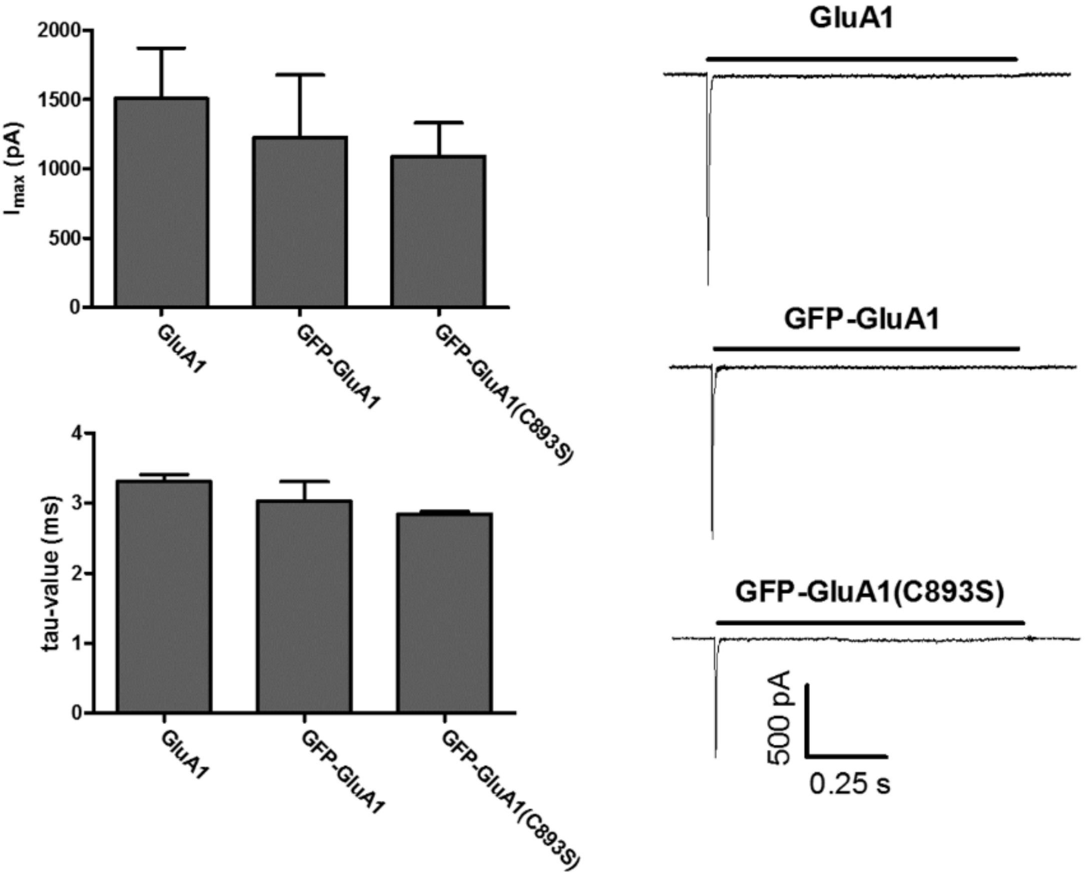

Figure A. The functional properties of GluA1 are not altered by the N-terminal GFP-tag or the cysteine to serine substitution at position 893. The maximal currents (I_{max}) evoked by 1 s application of 10 mM glutamate (indicated by the horizontal line) do not differ significantly between GFP-tagged GluA1 wt and C893S receptors as compared to myc-tagged GluA1 wt receptors (one-way ANOVA). Moreover, there is no significant difference in the tau-value (τ) of desensitization between the different receptor forms. The receptor currents were measured from transiently transfected HEK293 cells (n= 5-11 cells).

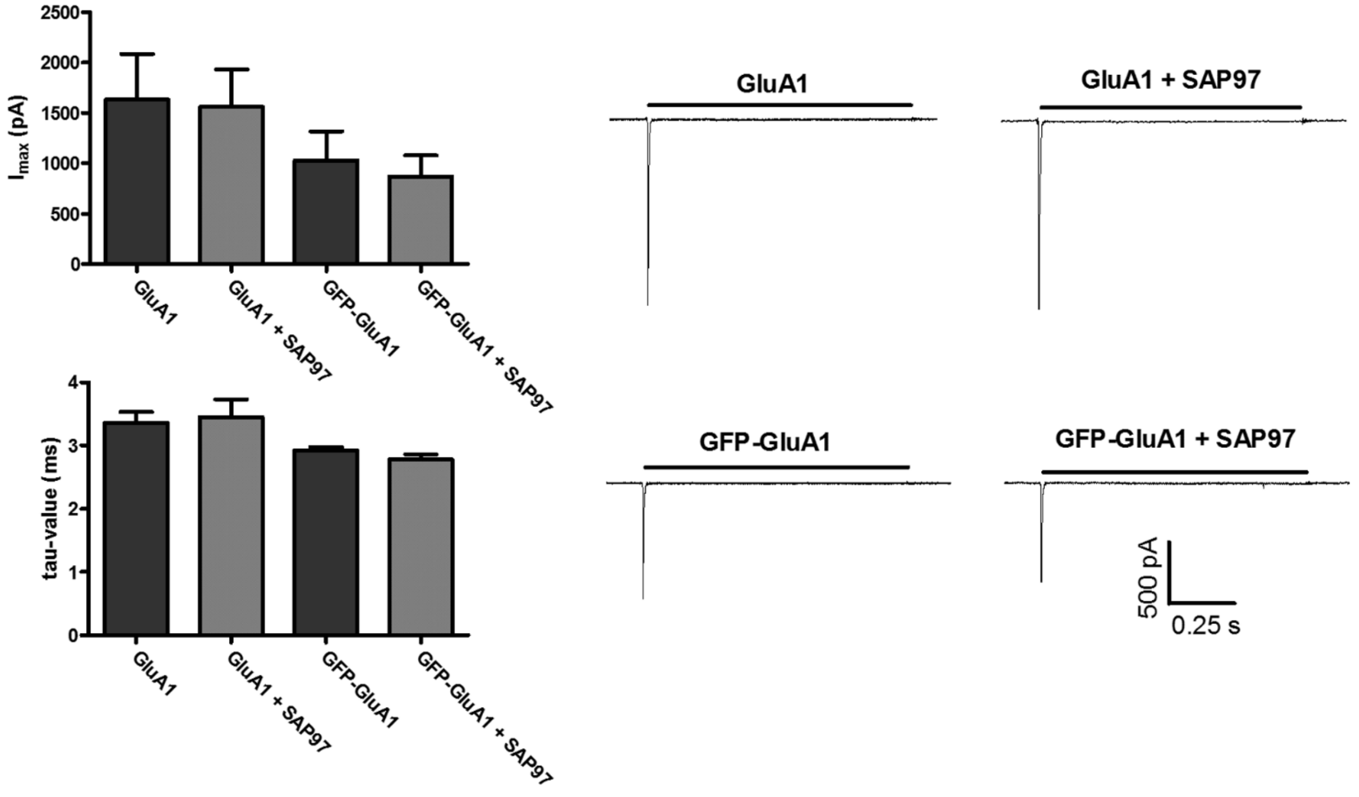

Figure B. The effect of SAP97 on GluA1 receptor currents. Co-expression with SAP97 does not alter the amplitude (I_{max}) or desensitization kinetics (τ) of glutamate responses by GFP- and myc-tagged GluA1 receptors (one-way ANOVA). The horizontal line indicates 10 mM glutamate application. The current responses were measured from transiently transfected HEK293 cells (*n*=10-16 cells).

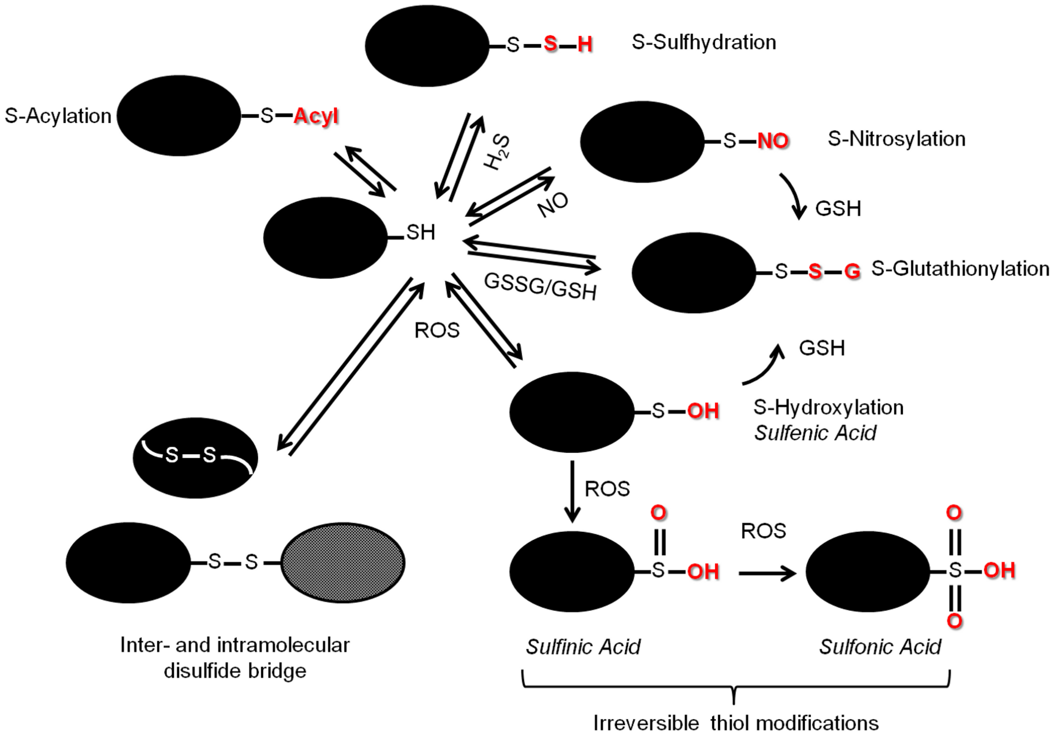

Figure C. Possible covalent thiol modifications of cysteine residues (-SH).

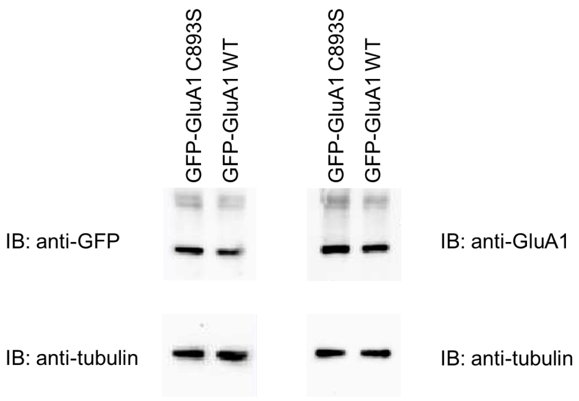

Figure D. GluA1 antiserum recognizes both wild-type GluA1 and GluA1 C893S point mutant. Western blot analysis of cell lysates prepared from HEK293 cells expressing GFP-tagged GluA1 wt or mutant GluA1. The upper left and right panels show the GluA1 bands detected by anti-GFP and anti-GluA1 antibodies, respectively, whereas the lower panels show the corresponding tubulin bands, serving as protein loading controls.
